# Concomitant primary ovarian paraganglioma neuroendocrinal differentiated urothelial tumor in a BOTOX-injected bladder: A case report

**DOI:** 10.1016/j.ijscr.2019.03.026

**Published:** 2019-03-28

**Authors:** M.A. Elbaset, Abdelwahab Hashem, M. Abd Elhameed, Ahmed S. El-Hefnawy

**Affiliations:** a Urology Department, Urology and Nephrology Center, Mansoura University, Egypt; b Pathology Department, Urology and Nephrology Center, Mansoura University, Egypt

**Keywords:** Case report, BCG, Paraganglioma, Neuroendocrine differentiation, Onabotulinumtoxin A (BTX) injection, Overactive bladder

## Abstract

**Introduction:**

There is no reported data for patients with malignant bladder Botox^®^ injection related outcomes. Herein, we reported effect of Botox^®^ injection in case of BCG cystitis. In addition, reporting of rare incidence of both primary neuroendocrine differentiation of bladder tumor and primary ovarian paraganglioma post cystectomy.

**Presentation of case:**

A-64 years old female presented with sever irritative lower urinary tract symptoms (LUTS) post vesical BCG installation for carcinoma in situ. Patient’s symptoms were relieved after Botox^®^ bladder injection after anticholinergics failure. One year later, she came back with hematuria. Cystoscopy showed nodular bladder lesion. Hypertension episodes were noticed during cystoscopic resection. Pathological examination showed presence of muscle invasive transitional cell carcinoma (TCC) with neuroendocrine differentiation. Patient underwent radical cystectomy and pathology was associated with incidentally discovered primary ovarian paraganglioma.

**Discussion:**

Intravesical BCG is a standard adjuvant treatment for carcinoma in situ with 75% induced cystitis as a local side effect. The International Bladder Cancer Group’s recommendation for BCG cystitis included many agents, these treatment options had very limited outcomes. The existence of paraganglioma in the female genital tract described rarely <1% in the vagina, uterus, vulva and ovary with only few reports. The same as paraganglioma small cell differentiation of vesical urothelial tumor is a rare entity with no reported cases of simultaneous occurrence and to differentiate each other as primary or metastatic.

**Conclusion:**

Bladder Botox^®^ injection could be offered as a treatment for overactive bladder-like symptoms in malignant cystitis. Neuroendocrine tumors are with a rare entity could be happened instantaneously in urogenital tract.

## Highlights

• Intravesical BCG is a standard adjuvant treatment for high risk NMIBC and CIS with 75% induced cystitis as a local side effect.
• BTX injection could be followed by improvements in BCG cystitis after failure of anticholinergics.
• Small cell tumors and paraganglioma are primary rare pathologies subtypes of neuroendocrine tumors can be happened coincidentally in two different organs.
• It is considered a critical point differentiating primary or metastatic in two different subgroups of tumors.

## 1 Introduction

Bladder cancer is the fourth most common tumor among males in the United States [1]. The most frequent symptom of bladder cancer is hematuria, whereas urgency, dysuria and bladder irritation may indicate muscleinvasive bladder tumors (MIBC) or carcinoma in situ [2]. Carcinoma in situ is treated initially by vesical BCG installation. Overactive bladder (OAB) like symptoms e.g.: (urgency, urgency incontienence, frequency) increased in patients with BCG treatment [3]. Antimuscrinics remain the first-line therapy treating overactive bladder symptoms specifically urgency. In patient diagnosed with OAB, after a trial of antimuscrinics without satisfactory improvement in symptoms in 3 months period, bladder injection of onabotulinumtoxin A (BTX) could be offered as the following step. However, there is no reported data for patients with malignant bladder BTX injection related outcomes.

Urothelial carcinomas (UC) may demonstrate a wide range of divergent histologic differentiation including squamous, glandular, micropapillary, sarcomatoid, small cell (neuroendocrine), clear cell, lymphepithelial, and plasmacytoid types [4]. Variants in the pathology report is a critical point as they affect staging, prognosis and therapeutic consequences. Small cell carcinoma differentiation of the urinary bladder is rare and represents <1% of all tumor diversity variant [5]. Current knowledge of this disease is limited and is based mainly on small series and case reports.

Paraganglioma in the ovary is uncommon accounting less than 1–2% of malignant ovarian neoplasms [6]. Only few cases reported for this rare pathology. To our knowledge, presence of two different primary tumors subtypes of neuroendocrine origin in two different pelvic organs is not reported before. So, we reported a complex case with simultaneous incidence of primary neuroendocrine differentiated urothelial tumor in the bladder and another primary ovarian paraganglioma post radical cystectomy in a patient with history of BCG cystitis managed by BTX injection with improved LUTS. The work has been reported in line with the SCARE criteria [7].

## 2 Case report

**Fig. 1:**
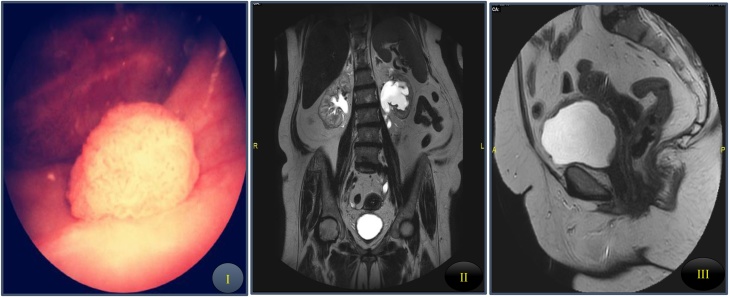
I. Cystoscopic examination revealed nodular mass at the trigone with smooth surface near left ureteric orifice. II and III. 3-Tesla MRI revealed average sized both kidneys with mild right hydronephrosis and left moderate hydronephrosis down to thickened bladder wall more at posterior bladder wall encroaching at both ureteric orifices. Bilateral external iliac lymphadenopathy and normal both ovaries.

**Fig. 2:**
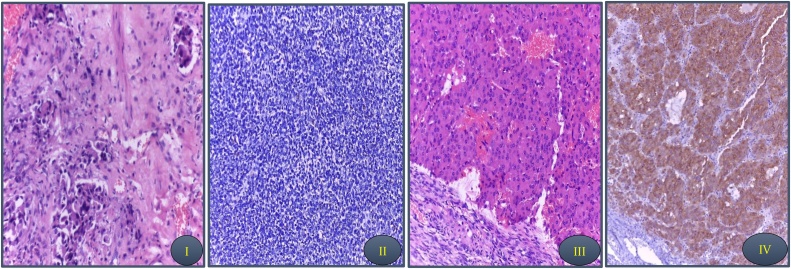
I and II bladder specimen, III and IV ovary specimen; I. Sheets of glandular structures of malignant transitional cells infiltrating lamina propria. The malignant cells exhibited marked anaplasia and pleomorphism. Foci of necrosis were evident. II. Pancytokeratin staining, neuroendocrine tumors with focal Chromogranin positivity and Synaptophysin involved in less than 10%. The adjacent mucosa was ulcerated. III. The left ovary revealed sheets of polygonal cells. The cells have abundant eosinophilic cytoplasm and rounded vesicular nuclei. IV. The left stroma is fine and vascular revealed negative pancytokeratin and diffuse positivity for Chromogranin and strong staining for Synaptophysin and inhibin.

A-64 year old female patient presented initially with dysuria for one month. She was suffering from hypertension controlled on different types of antihypertensive drugs with no attacks of headache, palpitation and sweating. She underwent diagnostic cystoscopy which revealed hyperemic velvety area at the bladder. Transurethral Resection of Bladder Tumor (TURBT) was done and pathology was carcinoma in situ. Patient received full course of vesical installation of BCG for 1 year (Six doses weekly as induction and nine doses monthly as maintenance treatment). No obvious complications were noticed during installation period. Follow up cystoscopies, urine cytology and radiology were free during BCG installation. However, patient developed sever irritative LUTS, in time of intravesical installation and post installation with poor improvement on anticholinergics. So, a decision was taken by bladder 100 IU BTX injection aiming to decrease symptoms bother. Bladder was inspected and biopsy was taken 2 weeks before injection with evidence of chronic cystitis. Follow up revealed improvement of suprapubic pain and LUTS. She had not attend for follow up for one year when she developed recurrent attacks of hematuria with developing attacks of headache, palpitation and sweating with no history of syncopal attacks during micturition. Outpatient cystoscopy showed nodular lesion at the trigone near left ureteric orifice with smooth outline (Fig. 1I). MRI for clinical staging revealed presence of diffuse thickening of the bladder base with bilateral external iliac lymphadenopathy and normal both ovaries (Fig. 1II and III). In addition, complementary bone scan was also done and was free. During resection hypertensive episodes reaching 220/120 mmHg were recorded. Pathology was high grade muscle invasive UC with neuroendocrine differentiation. Patient was planned for open radical cystectomy and ileal loop conduit. Notable bouts of hypertension on manipulation of the bladder were recorded intraoperatively. Patient passed smooth postoperative course stopped drugs of hypertension and discharged safely. Microscopic pathology came to be high grade UC muscle invasive of the bladder with neuroendocrine differentiation with positive staining for Pancytokeratin, Chromogranin and Synaptophysin and negative 15 lymph nodes. Also, incidentally primary left ovarian neuroendocrine tumor discovered with intensely staining for Chromogranin, Synaptophysin with negative Pancytokeratin/epithelial markers (Fig. 2).Two weeks following the surgery, the patient was tested with a 24-h urinary metanephrins and was within normal limits. Six month later patient underwent MRI and bone scan for follow up and was free with no evidence of local or distant recurrence with no history of headache or palpitation with still stoppage of antihypertensive drugs.

## 3 Discussion

Approximately 75% of patients with bladder cancer present with a disease confined to the mucosa (stage Ta, carcinoma in situ) or submucosa (stage T1). Carcinoma in situ is a flat high grade non-muscle invasive bladder cancer (NMIBC), without any treatment, approximately 54% of patients with carcinoma in situ progress to muscle invasive disease [8]. Intravesical BCG is a standard adjuvant treatment for high risk NMIBC and carcinoma in situ with 75% induced cystitis as a local side effect, usually self-limiting and rarely requires cessation of BCG [9]. Despite The International Bladder Cancer Group’s recommendation for BCG cystitis include the recommendation of oral oxybutynin, phenazopyridine, propantheline bromide or anti-inflammatory agents (NSAIDs), these treatment options are very limited and not backed up by high level evidence and more research is needed into the oncological outcomes of these therapies. In our case after the failure of conservative measures, we proceeded for BTX bladder injection followed by improvements in clinical symptoms. BTX contains a heavy chain that binds to the presynaptic terminal of the neuromuscular junction, and this then leads to internalization of the neurotoxic component, which is the light chain. The latter acts by inhibiting the release of acetylcholine from the presynaptic terminal of the motor end plate [10].

Histological differentiation in urothelial tumors is a relatively common phenomenon. In the United States 90% to 95% of bladder cancers are pure UC, and the remaining 5%–10% consist of UC with aberrant differentiation or non-urothelial carcinoma. This phenomenon was seen in presence of high-grade UC morphology. Prominently, most patients with histological divergent demonstrated extravesical disease at cystectomy. At the time of presentation, 57% were found to have locally advanced and/or metastatic disease. Small cell carcinoma differentiation presented in 0.5%–1.2% of total histological differentiation. Small cell carcinoma of the bladder usually affects patients over the age of 60 with a male predominance, besides, it could be classified into functional and non-functional tumors. Approximately 60% of patients have metastases at the time of diagnosis [11]. The frequent correlation of small cell carcinoma of the urinary bladder with urothelial carcinoma is suggesting that small cell carcinoma may originate from urothelial stem cell rather than a specific neuroendocrine precursor cell [12] Immunohistochemical stains show high positivity for Chromogranin, Synaptophysin, neuron-specific enolase, Keratin7, and epithelial membrane antigen (EMA).One of the differential diagnosis of small cell carcinoma is metastasis from another site. It is therefore important to differentiate paraganglioma from neuroendocrine tumors or differentiation. By the way, presence of keratin positive stains means presence of epithelial origin. Using this marker make the decision in our case to exclude metastasis between ovary and bladder.

The existence of paraganglioma in the female genital tract described rarely <1% in the vagina, uterus, vulva and ovary. All reported cases about ovarian paraganglioma were variable in age group ranging from young to postmenopausal women and variable size ranging from incidentally discovered to 22.5 cm, usually larger tumor were associated with lymph node metastasis or locally advanced disease. Main presentation was hypertension and rarely abdominal mass [13]. Differential diagnoses are based in immunohistochemical analyses. Paraganglioma exhibit positivity to neural markers such as Synaptophysin, Chromogranin, S-100 with low cytokeratin expression [13].

We reported a case of OAB like symptoms related to malignant cystitis, BTX could be used as an alternative to antimuscrinics with marked improvement of LUTS. Further studies should be conducted evaluating the efficacy and safety of BTX injection. Also, we described double primary rare pathologies subtypes of neuroendocrine tumors coincidental with each other post radical cystectomy.

## 4 Conclusion

Intravesical BTX injection could be offered as a treatment for OAB-like symptoms in malignant cystitis. Studying its safety and efficacy and its interaction in malignant bladder cells is mandatory. Neuroendocrine tumors are with a rare entity could be happened instantaneously in urogenital tract.

## Conflicts of interest

No conflict of interests.

## Sources of funding

No funds received.

## Ethical approval

Ethical approval has been exempted by our institution.(Not needed as it is case report).

## Consent

Written informed consent was obtained from the patient for publication of this case report and accompanying images. A copy of the written consent is available for review by the Editor-in-Chief of this journal on request.

## Author’s contribution

M.A. Elbaset collected the patient details and wrote the paper.

Abdelwahab Hashem made contributions to conception and design.

M. Abd Elhameed collected pathology images and revised them.

Ahmed S. Elhefnawy critically revised the article. All authors read and approved the final manuscript.

## Registration of research studies

Not done as it is case report.

## Guarantor

Ahmed S. Elhefnawy.

## Provenance and peer review

Not commissioned, externally peer-reviewed.
